# Multigene Expression Assay for Assessment of the Immune Status of Atlantic Salmon

**DOI:** 10.3390/genes11111236

**Published:** 2020-10-22

**Authors:** Aleksei Krasnov, Sergey Afanasyev, Stian Nylund, Alexander Rebl

**Affiliations:** 1 Nofima AS Osloveien 1, 1433 Ås, Norway; 2 Sechenov institute of evolutionary physiology and biochemistry Russian Academy Of Sciences, Torez 44, 194223 Saint-Petersburg, Russia; afanserg@mail.ru; 3 Pharmaq Analytiq, Thormoehlens Gate 53D, 5008 Bergen, Norway; stian.nylund@zoetis.com; 4 Leibniz Institute for Farm Animal Biology (FBN), Wilhelm-Stahl-Allee 2, 18196 Dummerstorf, Germany; rebl@fbn-dummerstorf.de

**Keywords:** Atlantic salmon, smolt, gene expression, immune competence

## Abstract

We report the development of a multigene gene expression assay on the BioMark HD platform for the evaluation of immune competence (ImCom) in farmed Atlantic salmon. The first version of the assay included 92 genes selected on the basis of transcriptome analyses in 54 trials that challenged the immune system; annotations were taken into account to represent the key pathways of innate and adaptive immunity. ImCom was tested on samples collected from seven independent projects. Fish were reared from the start feeding to eight months in the sea at eight units in different parts of Norway. Several tissues were analyzed. Linear discriminant analysis (LDA) showed that no more than 10 genes were required to separate groups, and a set of 46 immune genes was sufficient for any task. The second version of the assay was tested in the gills of two groups of high-performing healthy smolts and in groups with intermediate and high mortality rates (IM and HM, respectively). A set of 645 gill samples from clinically healthy Atlantic salmon was used as a reference. The IM group showed general suppression of immunity. All HM group salmon were above the threshold by the squared deviation from the reference. This group showed marked upregulation of genes involved in acute stress and inflammation: *mmp-9, mmp-13, hsp70, il-1b, lect2*, and *cathelicidin*. Further work will clarify the boundaries of the norm and explore various cases of impaired immunity.

## 1. Introduction

We present the development of a tool for assessment of the immune status of farmed Atlantic salmon. Infectious diseases are a major issue and the main source of losses in commercial aquaculture [1,2]. The ability of fish to combat pathogens is one of the key factors determining the success of salmon production. Assessment of immune status in fish is especially important before seawater transfer (SWT) and during the first several months in the sea when most disease outbreaks occur. High risks from transportation stress, rearrangement of osmoregulation, and adaptation to the marine environment with increased pathogen pressure are aggravated by immune suppression during smoltification [3], which is most likely associated with dramatic endocrine changes during this period [4,5], and continues for several months after SWT [6]. Fish that appear to perform equally and do not have visible health problems can have varying degrees of immune system competence (ImCom). Diagnosis of immune system competence can help to evaluate and mitigate risks. It can also contribute to the improvement of production protocols and nutrition and assist breeding for higher robustness and disease resistance. This study is a step toward the assessment of immune status based on a multigene expression assay.

Development of an ImCom assay involves both conceptual and technical problems. The selection of genes is complicated by the complexity and redundancy of the immune system; transcriptome studies have revealed high variation of immune responses even between similar challenge trials. Due to our extensive experience in this field, we consider the stability and reproducibility of expression profiles to be the main conditions for gene markers. We cannot select genes based only on levels of differential expression, since this would over-represent several functional groups, primarily antiviral and inflammatory responses, as many important immune genes do not show strong expression changes. Our assay design aims to cover the entire immune system and achieve a proper balance between different pathways and functional groups. For successful development, several more issues need to be addressed, including the minimum and optimal numbers of genes, the choice of tissues, the borders of the norm, and the character and acceptable levels of deviations. The immune status of fish can be compromised in various ways: suppression or unhealthy stimulation caused by infections or inflammatory agents—both issues can involve either the entire immune system or its specific parts. In addition, it may not be possible to establish a common standard of immune competence, and we have to determine whether the assay should be adapted for special cases and conditions, such as geographic areas, fish age and size or season. Definition of the norm requires extensive analyses of fish representing the full diversity of Atlantic salmon aquaculture. We report the first steps toward a diagnostic tool for the immune competence of smolts and growers of farmed Atlantic salmon in commercial aquaculture. A set of immune and stress genes was selected using Nofima’s microarray database STARS, which stores data from a large number of experiments and functional annotations of genes [7]. The multigene expression assay (ImCom) was developed on the Fluidigm Biomark HD platform and was tested in two phases: calibration and proof of evidence comparison of smolts with high, intermediate and poor performance.

## 2. Materials and Methods

### 2.1. Design of Multigene Expression Assay

The ImCom assay was developed on the BioMark HD platform (Fluidigm, München, Germany). The first version of the assay in a 96.96 format included four reference genes (*18s rRNA*, *b-actin, ef1a,* and *rps20* [8]) and 92 genes involved in immune and stress responses. Genes were selected based on expression profiles from previous experiments and functional annotations. Nofima’s bioinformatic system STARS stores the results of 115 experiments with Atlantic salmon and rainbow trout, 54 of which targeted the immune system. The experiments included challenges of fish and cell cultures with viruses, bacteria, parasites, and vaccination, and exposing them to inflammatory agents (Table 1). Data from these studies are stored in the National Center for Biotechnology Information Geo Omnibus. At the time of our ImCom design, more than 5000 of the 15k and 44k DNA oligonucleotide microarrays had been used. STARS stores primary and processed data: transcription signatures, lists of differentially expressed genes (DEGs) from each experiment, and contrasts–expression ratios of DEGs in pairwise comparisons (e.g., infected versus intact salmon, time-points, etc.). Genes are annotated with public (Gene Ontology–GO and Kyoto Encyclopedia of Genes and Genomes–KEGG) and custom vocabularies. Preliminary selection was based on the stability of responses (i.e., the proportion of similar experiments with differential expression). A second round of selection was used to ensure the coverage of immune pathways. To represent adaptive immunity, it was necessary to prioritize gene roles over expression data, since the transcriptional responses of these genes are less significant than those of genes involved in antiviral immunity and inflammation. The state of current knowledge was also considered; *ceteris paribus*, preference was given to well-characterized genes. Nine genes encoding chaperones, enzymes, and transcription factors involved in responses to stress and DNA damage were included in the assay because they are steadily co-activated with immune genes and most likely form an important part of defense against infections. The design used a combination of hypothesis-free and targeted approaches, although the contribution of the former was greater overall; genes without strong and reproducible transcription changes are useless for expression analyses, regardless of their roles and importance.

Primers adapted for the simultaneous detection of the various paralogs of the respective Atlantic salmon genes were designed using Pyrosequencing Assay Design software v.1.0.6 (Biotage, Uppsala, Sweden). For most genes, either the sense or antisense primer was placed on exon–exon boundaries. The assay-specific standard curves based on serial 10-fold dilutions starting from 1 × 10^2^–1 × 10^8^ copies of the individual amplicons [9] were generated using a LightCycler96 Real-Time PCR System (Roche, Basel, Switzerland). The efficiency of the primer pairs was determined with standard curves ranging from 90.7% to 110.3% (coefficient of determination R^2^ = 0.999). The list of genes and primers is in Supplementary Materials Table S1.

### 2.2. Fish Material

**Figure 1:**
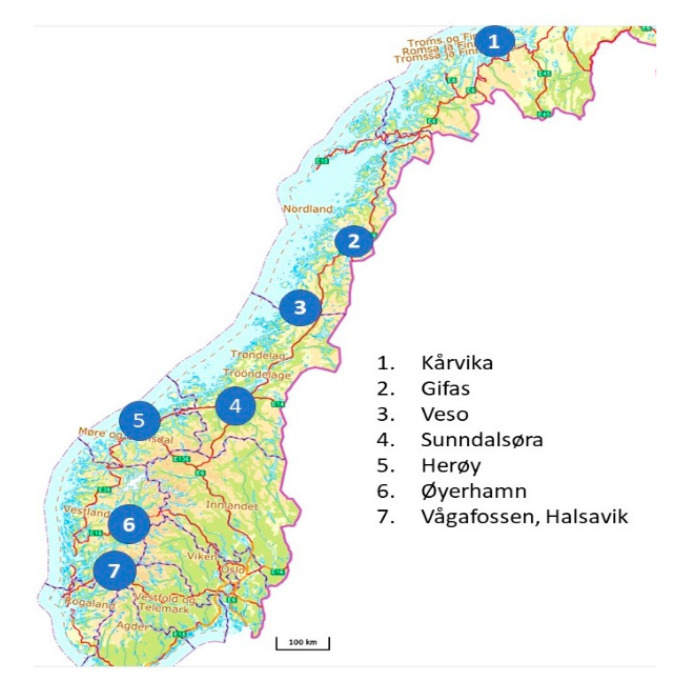
Location of aquaculture facilities where samples were collected.

The selection and composition of samples are presented in Table 2. We sought to represent the greatest variety of fish produced by Norwegian commercial aquaculture. The types of aquaculture facility (traditional flow through and recirculation land units, recirculating aquaculture system (RAS); large and small cage sea farms owned by the industry and research organizations) are listed in Table 2, and the geographic areas of Norway included in the study are shown in Figure 1. The production period ranged from the start of feeding to eight months in the sea. Sampling was performed within seven fully independent research projects approved by the Norwegian Food Safety Authority (approval codes 16,890, 11,814, 13,160, 8586, and 17,725) at eight sites located in different parts of Norway (Figure 1). All sampling procedures were carried out in accordance with guidelines from the Norwegian Food Safety Authority and the associated EU Directive 2010/63/EU guidelines for animal experiments. Samples of the head kidney, spleen, heart, dorsal fin, and gill were placed in RNALater (ThermoFisher Scientific, Waltham, MA, USA) and stored at −20 °C.

### 2.3. Analyses

For isolation of RNA, small tissue pieces (5–10 mg) were placed in tubes with 400 µL lysis buffer (Qiagen, Hilden, Germany) and steel beads, and 20 µL proteinase K (50 mg/mL), (Qiagen, Hilden, Germany) was added to each tube. Samples were shaken in a FastPrep 96 (MP Biomedicals, Eschwege, Germany) for 120 s at maximum speed, then centrifuged and incubated for 30 min at 37 °C. RNA was extracted by a Biomek 4000 robot (Beckman Coulter, Brea, CA, USA) using the Agencourt RNAdvance Tissue kit (Qiagen, Hilden, Germany). Concentration was measured with a NanoDrop One (ThermoFisher Scientific, Waltham, MA, USA), and integrity was assessed with an Agilent (Santa Clara, CA, USA) Bioanalyzer 2100. The extracted RNA (1 µL) was reverse-transcribed using the master mix (Fluidigm, München, Germany). The individual cDNA samples were adjusted at 10 ng in 5 μL. After addition of primers (100 µM) and the PreAmp master mix (Fluidigm, München, Germany), 12 pre-amplification cycles were run in a TAdvanced thermocycler (Biometra, Jena, Germany). The products were treated with exonuclease I (New England BioLabs, Ipswich, MA, USA) and diluted in an SsoFast EvaGreen supermix with Low ROX (Bio-Rad, Hercules, CA, USA) and a 20× DNA-binding dye sample loading reagent. The samples and primer mixes were loaded into the respective inlets of the integrated fluidic circuit (IFC) chips, primed in the BioMark IFC controller MX (48.48) or HX (96.96) (Fluidigm, München, Germany) and placed in the BioMark HD system (Fluidigm, München, Germany). qPCR was performed according to array-specific cycling programs. The raw qPCR results were retrieved with real-time PCR analysis software v. 3.0.2 (Fluidigm, München, Germany) and transferred to a relational database. The geometric means of *ef1a* and *rps20*, the two reference genes with stable expression, were used for calculation of ΔΔCt values.

### 2.4. Statistics

Statistical analyses (linear discriminant analysis (LDA), normality of distributions, and ANOVA, followed by post hoc tests and pairwise comparisons with *t*-test (*p* < 0.05) were performed using Statistica 13.

## 3. Results

### 3.1. Gene Expression—Calibration

Assessment of immune status required comparison of test samples with healthy Atlantic salmon, which showed high performance and were not known to be suffering from compromised immunity or any other adverse conditions. The first phase of the ImCom design aimed to answer several important questions, such as selection of tissues for diagnostics. The head kidney and spleen were included as the primary and secondary lymphatic organs of teleost fish [10,11]. The hearts of fish can develop strong immune responses to the viral pathogens [12]. The use of gills and dorsal fins allows simple and fast noninvasive sampling. In our transcriptome studies, gills have shown developmental expression changes in immune genes, which are overall smaller in comparison with lymphatic organs but are still relatively large [3]. We had no experience with transcriptome analyses in fins, but salmon skin is characterized by its high transcriptome complexity, strong responses to pathogenic bacteria and parasites, and large changes in gene expression during the sea phase [6,13].

**Figure 2:**
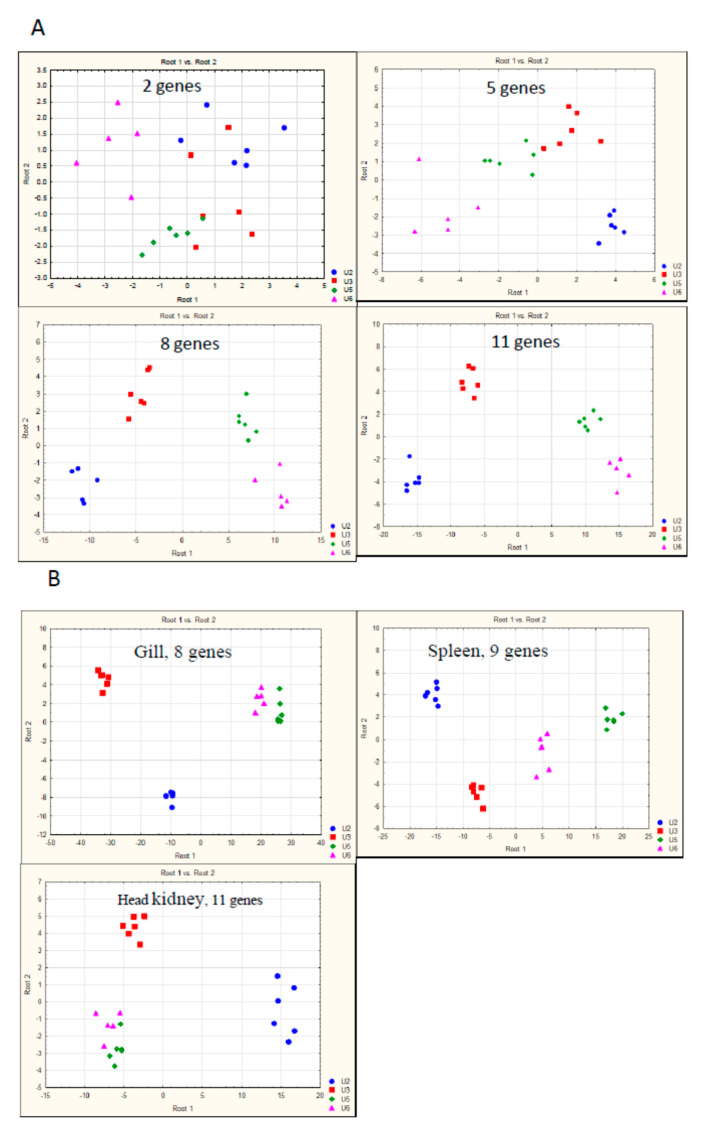
Linear discriminant analysis (LDA) of gene expression. Samples were collected at Sunndalsøra and Gifas research stations within the Korssting project (see Table 1). (**A**) Fin, separation of sampling groups with different numbers of genes. (**B**) Optimum separation of sampling groups by gene expression in the head kidney, gill, and spleen. U2, U3-10 and 31 days after end of stimulation with constant light; U5, U6–56 and 105 days after seawater transfer.

The qPCR analyses are usually carried out with small numbers of genes selected for specific cases and research tasks. It was not known whether a set of genes compiled to reflect the general state of the Atlantic salmon’s immune system could be used to separate any arbitrary groups (such as time-points, facilities, treatments) or how many genes would be required. We analyzed different sampling groups with LDA to address technical challenges: determining which tissues would work best for discrimination and how many genes were required. A typical result is presented in Figure 2. A cohort of Atlantic salmon was transferred from a land facility (Sunndalsøra) to a sea cage farm (Gifas). Samples of the spleen, gill, and dorsal fin were collected from smolts (ten and 31 days after end of stimulation with constant light—U2 and U3 in Figure 2) and growers (56 and 105 days after SWT—U5 and U6 in Figure 2). As in all other analyses, LDA began with two genes selected based on the greatest difference in expression, and continued with the addition of one gene at each step until the complete separation of the groups was achieved. In the dorsal fin, five was the minimum number of genes providing a vague resolution of the time-points (Figure 2A). The results improved with the addition of more genes and the optimum was reached with eleven genes. Similar results were produced in other tissues (Figure 2B), where eight to eleven genes were required. However, no clear separation of the last two time-points was achieved for the head kidney or gill. Similar conclusions were drawn from all LDA analyses: the external tissues were not inferior to the lymphatic organs, and all sampling groups could be separated using eight to eleven genes. LDA is a method designed to find an optimal solution, but not the best and only solution. In each run, it proposed many equally valid options with different combinations of genes, making it impossible ranking the genes by the diagnostic value and finding a universal core subset of immune genes. Consequently, the total number of genes in the ImCom assay should be several times higher than that mentioned above. In this phase (development and calibration of the assay), we used 92 immune and 4 reference genes. The smaller Biomark HD (48.48) significantly simplifies analysis and decreases its cost. We selected 46 immune genes by variance of expression, considering functional annotations (Supplementary Materials Table S1). The LDA of available samples suggested that this reduction was unlikely to affect the power of analyses. In addition to the immune genes, the second version of ImCom included two reference genes (*ef1a* and *rps20*) that showed high stability across all samples.

### 3.2. Reference Data Set and Assessment of Immune Status

Decision to continue analyses in only one tissue and to halve the number of genes allowed us to increase the number of samples, and a gill reference data set (GRDS) with 645 gill samples was compiled. Two simple metrics were tested. Mean deviation (MD) calculated as *∑n(xi – x̅i)/n*, (where *xi* and *x̅i* are, respectively, values in a sample and reference set and *n*—number of genes) shows overall expression changes (prevalence of up or downregulation). Mean squared deviation (MSD), or *∑n(xi − x̅i)^2^/n*, increases when the immune system is out of balance (many up- and down-regulated genes). In the GRDS, the MD followed a normal distribution (Kolmogorov–Smirnov test), while the MSD did not fit any of the commonly used distributions. We therefore determined fifth and tenth percentile thresholds empirically.

**Figure 3:**
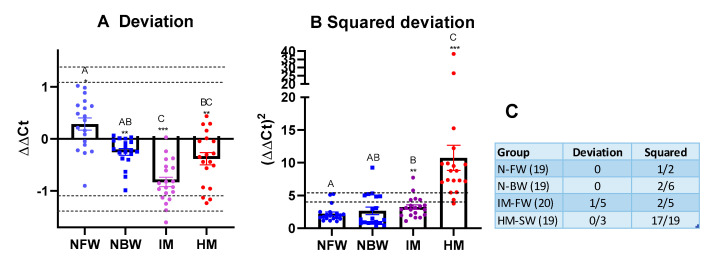
Comparison of smolts with different performance with the Gill Reference Data Set (GRDS). Each mark (circle, square or rectangle) corresponds to an individual. (**A**) Mean deviation; (**B**) squared deviation. Columns not sharing common letters are significantly different (ANOVA, Tukey’s test, *p* < 0.05). Asterisks mark difference from GRDS by the mean values: * < 0.05, ** < 0.01, and *** < 0.001. Dotted lines denote fifth and tenth percentile thresholds. (**C**) Numbers of fish exceeding thresholds (fifth/tenth).

Proof-of-evidence analyses were carried out on four groups of smolts produced by the industry, which had known performance levels: normal smolts maintained in fresh and brackish water (NFW and NBW, respectively) and smolts with intermediate and high mortality (IM and HM, respectively). In the IM group, there were 20 fish and 19 fish in each of the other three groups. The MD of each group differed significantly from that of the GRDS (Figure 3A). A slight overall upregulation of immune genes was observed only in the NFW, and the IM group showed the strongest downregulation (approximately 1.8-fold). No individual fish exceeded the upper percentile thresholds, while suppression was found in five and three individuals from the IM and HM groups, respectively (Figure 3C). In comparison with GRDS, MSD increased significantly in both of the poor performing groups: IM and especially HM (Figure 3B). The numbers of fish with unbalanced expression of immune genes were noticeably higher in the batch with high mortality: all fish exceeded the tenth percentile threshold, and 17 of 19 were above the fifth percentile.

**Figure 4:**
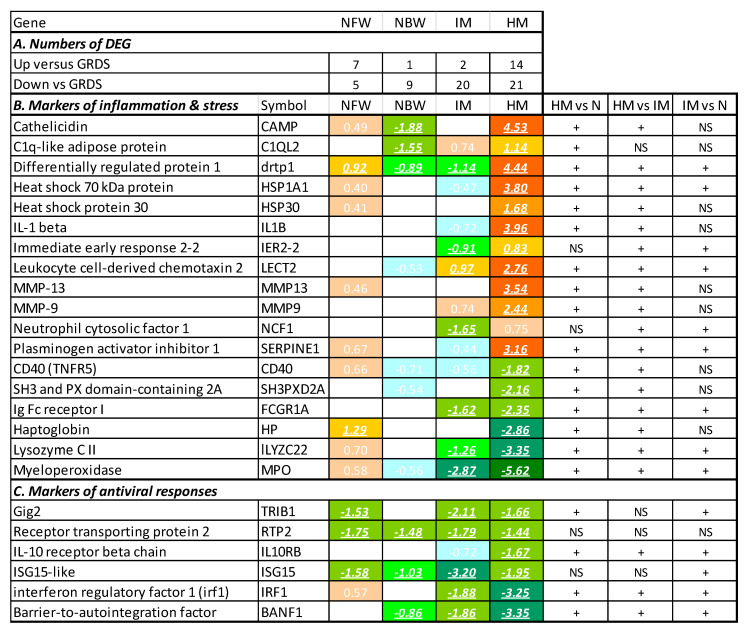
Differentially expressed genes (DEGs). (**A**) Numbers of genes with expression differences versus Gill Reference Data Set (GRDS). (**B**,**C**) Individual genes. The gene expression data are the differences of means between the smolt groups and the GRDS (ΔΔCt). DEGs are indicated with bold italics underlined. Differences from smolt batches with normal performance are indicated with +; NS—not significant.

Differentially expressed genes (DEGs) were selected by the same criteria applied in the microarray analyses (a difference of ΔΔCt > 0.8, or approximately 1.75-fold, and *p* < 0.05). In comparison with the GRDS, the numbers of DEG increased from 10 and 12 in two groups of normal smolts to 22 and 35 in smolts with intermediate and high mortality, respectively (Figure 4A). The smolt batch with high mortality showed upregulation of the same panel of genes that has been associated with acute inflammation and stress in our studies with transcriptome analyses (Figure 4B). These are *matrix metalloproteinases 9* and *13*, which degrade extracellular proteins, and proteinase inhibitor *serpine*, chemokine *lect2*, transcription factor *ier2*, antimicrobial peptide *cathelicidin*, *heat shock proteins 30 kda* and *70 kda*, and an uncharacterized gene denoted as *c1q-like adipose protein*. Unlike these genes, an emblematic mediator of inflammation (*il1b*) was included in the assay only by functional annotation. Overall expression of immune genes in HM slightly decreased (Figure 3A); downregulation was observed in a number of genes encoding effectors and the lymphocyte-specific proteins. Of note was the low expression of *myeloperoxidase (mpo)* in both smolt batches with increased mortality. Downregulation of *mpo* and the large group of innate antiviral response genes [14] observed here in all smolt batches (Figure 4C) was reported as a hallmark of immune suppression during smoltification [3].

## 4. Discussion

The discovery of significant downregulation of the immune genes in smolts and growers indicates a potential issue that could affect the commercial aquaculture of Atlantic salmon. Immune suppression may increase the risk of infectious diseases, especially during first several months in the sea, although at present we do not know the threshold at which the suppression becomes dangerous. The quality and robustness of salmon smolts are critically important for the success of aquaculture. Supplementation of currently used tests for osmoregulation with the evaluation of immune competence could be a significant benefit for the industry. Development of such diagnostic tools is a complicated task. Several hundred genes are involved in immune responses and defense against pathogens in Atlantic salmon. Consistent downregulation of immune genes during smoltification has been shown in a suite of studies. Because the sets of differentially expressed genes vary, immune status cannot be assessed by qPCR analyses of a small set of genes. Despite successful results from transcriptome analyses in farmed Atlantic salmon (comparison of several industrial sites with RNA-seq [15]), this method is unsuitable for routine diagnostics in large numbers of field samples. Multigene expression assays close the gap between qPCR and microarrays or RNA-seq and are attractive possibilities for fish immunology and aquaculture research. In this study, we used BioMark HD, which has produced impressive results in the ecoimmunology of Pacific salmon [16,17]. An important advantage of this system is flexibility: gene sets can be changed without any limitations. The goals of this study were to present the principles and demonstrate the potential of multigene expression assays for the assessment of immune competence. Appropriate selection of a representative set of immune genes is of critical importance and, in this respect, the microarray database containing accumulated data for Atlantic salmon during the last decade was useful. The ImCom assay should include genes with strong and consistent differential expression and key immune roles. These conditions can be controversial, since many well-explored and annotated genes do not show strong transcriptional regulation in Atlantic salmon. Therefore, both functional annotation and empirical data should be considered. The choice of candidate gene markers based on large expression changes in one or only a few studies can be misleading. Based on our experience in immunogenomics, we became convinced that reproducibility is the main criterion for selection of gene markers. Fish develop divergent transcriptome responses even in standard trials, and only a small panel of immune genes have shown relatively reproducible expression profiles. As expected, our results confirmed that discrimination of salmon batches cannot be achieved with qPCR analyses of limited sets of genes. An assay of 46 carefully selected immune genes is most likely sufficient for most diagnostic tasks, but it is difficult to predict which genes will be useful in each case.

We anticipated three types of compromised immunity in Atlantic salmon smolts and growers: (i) coordinated changes with the prevalence of downregulation (immune suppression) or upregulation (infection, inflammation), (ii) loss of balance (many genes deviating from the norm in both directions), and (iii) problems associated with expression changes in one or a few genes. The first two cases are diagnosed with integrative metrics that use all results with equal inputs from all individual genes. We are aiming at the practical application of ImCom; therefore, it was inspiring to find that simple metrics with clear biological interpretation achieved sufficient separation of smolt batches with different performance levels. In this pilot study, the MD (average deviation from the Gill Reference Data Set) was instrumental for comparison between the groups, as it is less sensitive at the individual level. The batch with intermediate mortality showed a strong tendency towards immune suppression: twenty downregulated and only two upregulated genes. Squared deviation (SMD) detected batches and individuals with apparently compromised immunity. Inspection of DEGs did not add to the interpretation of results for the IM group but was informative for the smolts with high mortality. It suggested acute inflammation and stress, together with the suppression of other immune functions. Upregulated genes are known for their strong association with diseases and pathology.

In two batches of normal smolts, 8 out of 38 fish exceeded the tenth percentile, and 3 of them were beyond the fifth percentile. These thresholds were set only for demonstration purposes and at present do not have any biological meaning. Expansion of the reference data set and determination of the boundaries of the norm will be the main tasks required for further development of diagnostics, which will need to include multiple analyses of healthy salmon and fish with different health problems. We used the reference data set that encompassed the period from the start feeding to eight months in the sea. A common quality standard is convenient from a practical point of view. However, considering large developmental changes, in the future, it may be useful to adjust the norm for different stages of salmon life. Fine-tuning may be required for other factors including the seasons, geographic zones and the genetic background of Atlantic salmon. The ImCom assay developed in this study can be used immediately and will continually improve as data and experience grow.

## 5. Conclusions

In this paper, we reported the design of an assay and procedures for data management and computations. The study produced promising results and can be regarded as a milestone in the development of tools for the assessment of immune status in Atlantic salmon. We are aware that the work is in its early phase and certain issues need to be resolved in the future. The primary tasks will include the accumulation of data for healthy, high-quality salmon and fish with different health problems and the determination of the norm and deviations.

## Figures and Tables

**Table 1:** Experiments used for selection of genes.

Type	Experiments	Contrasts
All	115	560
Immune	54	274
Bacteria	11	54
Viruses	24	102
Parasites	12	50
Vaccines	6	26
Stress	11	76

**Table 2:** Samples used for multigene expression analyses.

Project	Site	Time Points	Format	Age	Facility	Tissue	No. of Samples
Korssting	Sunndalsøra	1	96	Smolt	Land FT ^1^	Fin, gill, head, kidney, spleen	24
	Gifas	3	96	Postsmolt	Sea cages	Fin, gill, head kidney, spleen	72
Progress	Kårvika	3	96	Parr, smolt	Land FT	Head kidney, spleen, gill	96
ImCom: Animal Health	Veso	3	96	Parr, smolt	Land FT	Heart, spleen	160
ImCom: Mowi	Vågafossen	2	96	Parr, smolt	Land RAS ^2^	Fin, gill, spleen	72
	Herøy	2	96	Postsmolt	Sea cages	Fin, gill, spleen	72
	Øyerhamn	2	96	Parr, smolt	Land FT	Fin, gill, spleen	72
	Halsavik	2	96	Postsmolt	Sea cages	Fin, gill, spleen	72
Benchmark	Sunndalsøra	4	48	Parr, smolt	Land FT, RAS	Gill	194
	Gifas	1	48	Postsmolt	Sea cages	Gill	160
Farmwell	Sunndalsøra	1	96	Smolt	Land FT, RAS	Gill	72
Pharmaq Analytiq	Various	1	48			Gill	80

^1^ FT—flow-through facility; ^2^ RAS—recirculation aquaculture system.

## Supplementary Materials

The following are available online at https://www.mdpi.com/2073-4425/11/11/1236/s1, Table S1: ImCom assay.
